# Apoptotic Engulfment Pathway and Schizophrenia

**DOI:** 10.1371/journal.pone.0006875

**Published:** 2009-09-01

**Authors:** Xiangning Chen, Cuie Sun, Qi Chen, F. Anthony O'Neill, Dermot Walsh, Ayman H. Fanous, Kodavali V. Chowdari, Vishwajit L. Nimgaonkar, Adrian Scott, Sibylle G. Schwab, Dieter B. Wildenauer, Ronglin Che, Wei Tang, Yongyong Shi, Lin He, Xiong-jian Luo, Bing Su, Todd L. Edwards, Zhongming Zhao, Kenneth S. Kendler

**Affiliations:** 1 Department of Psychiatry and Virginia Institute for Psychiatric and Behavior Genetics, Virginia Commonwealth University, Richmond, Virginia, United States of America; 2 Department of Human and Molecular Genetics, Virginia Commonwealth University, Richmond, Virginia, United States of America; 3 The Department of Psychiatry, The Queens University, Belfast, Northern Ireland; 4 The Health Research Board, Dublin, Ireland; 5 Washington VA Medical Center, Washington, D. C., and Georgetown University School of Medicine, Washington, D. C., United States of America; 6 Department of Psychiatry, University of Pittsburgh School of Medicine and Graduate School of Public Health, Pittsburgh, Pennsylvania, United States of America; 7 Western Australian Institute for Medical Research, Centre for Medical Research, University of Western Australia, Perth, Australia; 8 Centre for Clinical Research in Neuropsychiatry, School of Psychiatry and Clinical Neurosciences, University of Western Australia, Perth, Australia; 9 Bio-X Center, Shanghai Jiao Tong University, Shanghai, People's Republic of China; 10 State Key Laboratory of Genetic Resources and Evolution, Kunming Institute of Zoology, Chinese Academy of Sciences, Kunming, People's Republic of China; 11 Miami institute of human genetics, University of Miami, Miami, Florida, United States of America; 12 Departments of Biomedical Informatics and Psychiatry, Vanderbilt University School of Medicine, Nashville, Tennessee, United States of America; Tokyo Medical and Dental University, Japan

## Abstract

**Background:**

Apoptosis has been speculated to be involved in schizophrenia. In a previously study, we reported the association of the *MEGF10* gene with the disease. In this study, we followed the apoptotic engulfment pathway involving the *MEGF10*, *GULP1*, *ABCA1 and ABCA7* genes and tested their association with the disease.

**Methodology/Principal Findings:**

Ten, eleven and five SNPs were genotyped in the *GULP1*, *ABCA1* and *ABCA7* genes respectively for the ISHDSF and ICCSS samples. In all 3 genes, we observed nominally significant associations. Rs2004888 at *GULP1* was significant in both ISHDSF and ICCSS samples (*p* = 0.0083 and 0.0437 respectively). We sought replication in independent samples for this marker and found highly significant association (*p* = 0.0003) in 3 Caucasian replication samples. But it was not significant in the 2 Chinese replication samples. In addition, we found a significant 2-marker (rs2242436 * rs3858075) interaction between the *ABCA1* and *ABCA7* genes in the ISHDSF sample (*p* = 0.0022) and a 3-marker interaction (rs246896 * rs4522565 * rs3858075) amongst the *MEGF10*, *GULP1* and *ABCA1* genes in the ICCSS sample (*p* = 0.0120). Rs3858075 in the *ABCA1* gene was involved in both 2- and 3-marker interactions in the two samples.

**Conclusions/Significance:**

From these data, we concluded that the *GULP1* gene and the apoptotic engulfment pathway are involved in schizophrenia in subjects of European ancestry and multiple genes in the pathway may interactively increase the risks to the disease.

## Introduction

Schizophrenia is a complex psychiatric disorder with strong genetic influences. Recently, many genes have been identified as potential susceptibility candidates [8], [10], [55], [56]. In a recent study, we reported the association of *MEGF10* with schizophrenia in our Irish family and case-control samples [7]. *MEGF10* is the human ortholog of the draper (*DRPR*) and cell death abnormal 1 (*CED-1*) gene in *Drosophila melanogaster* and *Caenorhabditis elegans* respectively [62]. In both *Drosophila* and *C. elegans*, *DRPR* and *CED-1* are key components of phagocytosis functioning in the clearance of apoptotic cells [23], [67]. In multicellular organisms, phagocytosis is a crucial process in development and in the innate immune system [17], [26]. In addition to its role in the clearance of apoptotic cells, *DRPR* plays a critical role in axon pruning and degenerating neurons [1], [30]. In *C. elegans*, *CED-1* mutations cause abnormal axon patterns and commissure branching [51].

In *C. elegans*, two parallel pathways are involved in the clearance of apoptotic cells. One involves the *CED-2*, *CED-5* and *CED-12* genes, which activate GTPase *CED-10* and trigger rearrangement of cytoskeleton during phagocytosis. The other involves the *CED-1*, *CED-6* and *CED-7* genes. *CED-1* is a membrane protein that senses cell death signals and recognizes neighboring cell corpses. It clusters at the phagocytic cups and initiates pseudopod extension. *CED-6* is an adaptor protein for *CED-1*, helping to control the delivery of vesicles to phagocytic cups and phagosomes. *CED-7* works with *CED-1* in recognizing engulfment signals of cell corpses. The detail functions of these genes are reviewed recently [12], [24], [32].

The apoptotic engulfment pathways are evolutionarily conserved and it is believed that they function similarly in higher organisms, including humans [24]. In the last several years, orthologs of several components of these pathways have been identified in mammals [28], [39], [63]. In humans, *MEGF10* and *GULP1* have been determined to be orthologs of *CED-1* and *CED-6* respectively [16], [28], [59]. For *CED-7*, two human genes, *ABCA1* and *ABCA7*, are proposed as orthologs [16], [19], [39]. Based on our study of the *MEGF10* gene and its function in apoptosis, we hypothesize that the apoptotic engulfment pathways are involved in the etiology of schizophrenia. To test the hypothesis, we conducted association studies of *GULP1*, *ABCA1* and *ABCA7*, located at chromosomes 2, 9 and 19 respectively, with the Irish study of high density schizophrenia families (ISHDSF) and Irish case-control study of schizophrenia (ICCSS) samples and followed up these analyses with targeted replication in multiple independent samples. In this article, we report the results from these studies.

## Results

### Association analysis

#### The *GULP1* gene

The *GULP1* gene was the first gene in the CED pathway we analyzed in this study. As in our previous studies [4]–[9], the ISHDSF sample was used as a screening sample and the ICCSS was used to follow up as a replication sample. For the 10 SNPs typed in the ISHDSF sample, multiple markers reached nominal significance by the pedigree disequilibrium test (PDT) [34] (Table 1). Subsequently, we typed the same SNPs in the ICCSS sample. Two SNPs, rs2004888 and rs4522565 were nominally significant, and another SNP, rs10469735, was significant at a trend level. Rs2004888 was the only marker that was significant in both ISHDSF and ICCSS sample, and the associated allele, T, was the same in the two samples. Overall, the 10 SNPs typed in *GULP1* gene have similar frequencies and LD structure in both ISHDSF and ICCSS samples (see Table S1, Figure S1 in supplementary materials).

**Table 1:** Single marker association analyses of the GULP1 gene.

Marker	ISHDSF									ICCSS			
	Transmitted Allele	Z	P*	Trio-T	Trio-NT	AffSib	UnafSib	Allele Freq	OTR	P*	Case Freq	Ctrl Freq	OR
rs6718697	A	2.10	**0.0356**	140	134	1195	1149	0.857	1.09	0.1395	0.889	0.872	1.18
rs9808557	C	2.34	**0.0193**	126	120	1120	1072	0.816	1.10	0.1381	0.854	0.835	1.16
rs10469735	T	1.85	0.0649	144	137	1175	1134	0.853	1.09	0.0659	0.889	0.868	1.22
rs2004888	T	2.64	**0.0083**	140	132	1196	1143	0.856	1.11	**0.0437**	0.889	0.866	1.25
rs6753371	A	1.99	**0.0468**	86	79	654	620	0.511	1.15	0.4524	0.496	0.482	1.06
rs4413123	G	2.19	**0.0282**	89	82	721	673	0.547	1.16	0.7409	0.463	0.457	1.02
rs4522565	T	0.43	0.6700	136	134	1142	1134	0.830	1.02	**0.0062**	0.873	0.838	1.33
rs6714454	A	1.77	0.0761	4	3	42	15	0.028	4.81	0.9040	0.020	0.020	1.03
rs7595327	G	0.82	0.4134	131	129	1075	1065	0.792	1.03	0.1278	0.833	0.812	1.16
rs8273	C	0.66	0.5090	134	134	1142	1138	0.872	1.00	0.7725	0.871	0.875	0.97

Trio-T, trio transmitted; Trio-NT, trio not transmitted; Affsib, affected sib; UnafSib, unaffected sib; Freq, frequency; Ctrl, control; OR, odds ratio; OTR, odds of transmission ratio.

* P values ≤0.05 are in bold.

#### The *ABCA1* and *ABCA7* genes

We typed 11 and 5 SNPs for the *ABCA1* and *ABCA7* genes to test whether these genes in the phagocytosis pathway may impact on risk for schizophrenia. Rs4149324 in *ABCA1* was nominally associated with the disease in the ICCSS sample (*p* = 0.0258) and rs2242436 in *ABCA7* was significant in the ISHDSF sample (*p* = 0.0013) (supplementary Table S2). Like the *GULP1* gene, markers in *ABCA1* and *ABCA7* genes have similar frequencies and LD structure in the two Irish samples (Table S1 and Figure S1).

### Gene-gene interaction in the CED pathway

**Figure 1:**
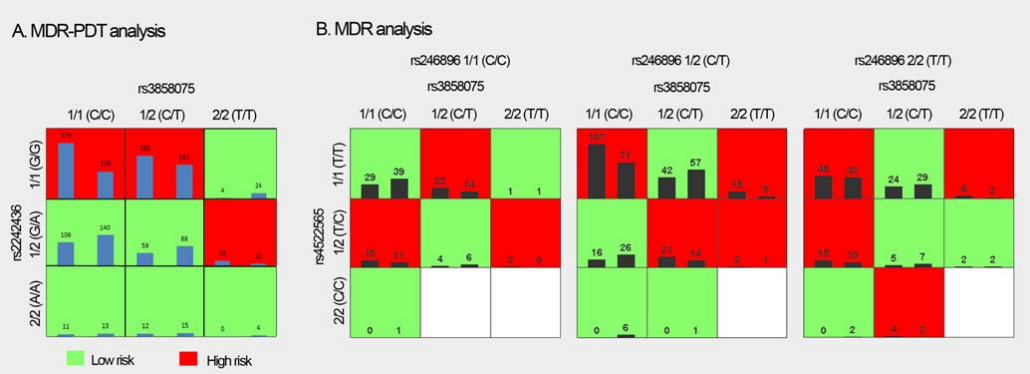
MDR-PDT and MDR analysis of the *MEGF10, GULP1, ABCA1* and *ABCA7* genes in the ISHDSF (A) and ICCSS (B) samples. For each cell, the count of affected individuals was plotted as the left bar, the count of unaffected was plotted as the right bar. The risk genotype combinations, defined as ratio of affected/unaffected in each cell, were highlighted.

To test whether genes interact to increase risk for schizophrenia, we examined gene-gene interaction by the multifactor dimensionality reduction pedigree disequilibrium test (MDR-PDT) [35] and the multifactor dimensionality reduction method (MDR) [46] for the ISHDSF and ICCSS samples respectively. In both samples, 36 SNPs typed in the *MEGF10* (10 markers), *GULP1* (10 markers), *ABCA1* (11 markers) and *ABCA7* (5 markers) gene were included in the analyses. We limited our search to 2- and 3-marker interactions. In the MDR-PDT analyses of the ISHDSF sample, we found a significant 2-marker interaction (*p* = 0.011): SNPs rs3858075 in the *ABCA1* gene and rs2242436 in the *ABCA7* gene interact with each other to increase risk of the disease. In the model, 3 genotype combinations of rs3858075 and rs2242436 (C/C-G/G, C/T-G/G and T/T-G/A) were overrepresented in the affected individuals (Figure 1). The overall OR of these groups were 26.2, 95% CI 3.2–210.8, *p* = 0.002 (Table 2). In the analyses of the ICCSS sample, we found a significant 3-marker interaction (*p* = 0.006). These 3 markers come from the *GULP1* (rs4522565), *ABCA1* (rs3858075) and *MEGF10* (rs246896) genes. In this model, the most abundant risk genotype group is T/T-C/C-C/T for markers rs4522565-3858075-rs246896 (Figure 1). None of these markers had significant main effects in our regression analysis. In contrast, the 3-marker interaction term was significant with an OR of 1.31, 95% CI 1.03–1.7, *p* = 0.0120 (Table 2). SNP rs3858075 in the *ABCA1* was involved in both the 2-marker and 3-marker interactions observed in the two samples. No gene-gene interaction analyses were conducted for other samples since they were typed only for rs2004888.

**Table 2:** Effect estimates of the gene-gene interaction.

Sample	Regression Term	Odds Ratio	95% CI	P-value
ISHDSF	rs2242436	0.079	0.011–0.622	**0.0159**
	rs3858075	0.179	0.033–0.984	**0.0478**
	rs2242436* rs3858075	26.168	3.249–210.758	**0.0022**
ICCSS	rs3858075	0.983	0.821–1.071	0.1520
	rs4522565	0.897	0.802–1.003	0.1580
	rs246896	0.947	0.813–1.103	0.4580
	rs3858075* rs4522565* rs246896	1.308	1.025–1.669	**0.0120**

### Replication of rs2004888

Based on the results of the *GULP1* gene, we selected rs2004888 for replication in other samples. In addition to the ICCSS, we typed this marker in the German, Pittsburgh, Shanghai and Kunming samples. As shown in Table 3, of the 5 replication samples, only ICCSS and Kunming samples were nominally significant, but the associated alleles were different in these two samples. When the 3 Caucasian replication samples were combined (3139 subjects, 1378 affected), the result was very significant (*p* = 0.0003). For the two Chinese samples, the Shanghai sample had the same allele overrepresented in the affected individuals as the Caucasian samples. But the Kunming sample was in the opposite direction. When the Chinese samples combined (cases  = 1458, controls  = 1740), the results were not significant. When all 5 replication samples were combined (6131 subjects, 2836 affected), the association became weaker (*p* = 0.0057, Table 3) due to the Chinese samples. Given our limited testing in these genes, the *p* value for the Caucasian replication samples survived Bonferroni correction for experiment-wide significance. But when the 2 Chinese samples were added, while the overall *p* value was nominally significant, it would not survive experiment-wide correction. We verified these results by meta-analysis. When all samples were included in the meta-analyses, rs2004888 was significantly associated with the disease (OR = 1.09, 95% CI 1.02–1.16, *p* = 0.011). While the meta-analysis results for all replication samples were not significant (OR = 1.06, 95% CI 0.98–1.13, *p* = 0.152), the results for Caucasian samples were significant (OR = 1.15, 95% CI 1.03–1.29, *p* = 0.0140). These results were consistent with the combined analyses.

**Table 3:** Replication of rs2004888*.

Sample	Case Cnt (Freq)	Ctrl Cnt (Freq)	T	NT	OR (OTR)	P
ICCSS	1380 (0.889)	1319 (0.866)	–	–	1.25	**0.0437**
Pittsburgh	–	–	331 (0.836)	325 (0.821)	(1.11)	0.4383
German	–	–	457 (0.856)	443 (0.830)	(1.22)	0.2265
Shanghai	1693 (0.928)	1662 (0.915)	–	–	1.22	0.1300
Kunming	885 (0.922)	1458 (0.943)	–	–	0.71	**0.0364**
Caucasian samples	1380 (0.889)	1319 (0.866)	788 (0.847)	768 (0.826)	1.25 (1.17)	**0.0003**
Chinese samples	2578 (0.926)	3120 (0.928)	–	–	0.97	0.7625
All replication samples	3958 (0.913)	4439 (0.909)	788 (0.847)	768 (0.826)	1.05 (1.17)	**0.0057**
All samples	3958 (0.913)	4439 (0.909)	928 (0.855)	1345 (0.846)	1.05 (1.07)	**0.0030**

* Cnt, count; ctrl, control; Freq, frequency; T, transmitted; NT, not transmitted; OTR, odds of transmission ratio.

## Discussion

While there is compelling evidence that genetic factors are involved in the etiology of schizophrenia, the involvement of specific genes and variants remains elusive. Most recent efforts to identify the risk genes focus on genome-wide association followed by targeted replications. Using this strategy, some genes have been identified as promising candidates in very large samples [42], [43]. Our group has taken a different approach, focusing on the clearly defined biological pathways and interactions. Using this approach, we identified that both *IL3* and *CSF2RB* are involved in the disease [4], [6], and independent studies [27], [38], [60] provide further support that *CSF2RB*, *CSF2RA* and *IL3RA*, the common and interleukin specific subunits of *IL3* and *CSF2* receptors, may also play an etiological role. Collectively, these studies support a role for the *IL3/CSF2* pathway in schizophrenia. This study follows the same strategy and extends our finding of *MEGF10* involvement in schizophrenia [7]. *MEGF10* is the human ortholog of *CED-1*, a key component of apoptotic engulfment pathway in *C. elegans*. Studies have shown that this phagocytic pathway is evolutionarily conserved, and multiple orthologs of CED genes have been identified in *Drosophila* [1], mouse [2], rat [36] and human [28], [39], [62]. Furthermore, protein products of these ortholog genes perform similar biological functions in the same pathway [17], [23], [26], [28], [39], [58], [59], [62], [63], [68], [69]. To test whether other CED orthologs in human are involved in schizophrenia, we conducted association analyses of the *GULP1*, *ABCA1* and *ABCA7* genes that are the human orthologs of the *CED-6* and *CED-7* genes. In this study, we found nominal associations in all 3 genes. Most importantly, rs2004888, a marker in the *GULP1* gene, was consistently significant (*p* = 0.0003) in combined Caucasian replication samples (3139 subjects, 1378 affected). In addition, we found significant gene-gene interactions in both our ISHDSF and ICCSS samples that involve all known human orthologs (*MEGF10*, *GULP1*, *ABCA1* and *ABCA7*) of the *CED-1*, *CED-6* and *CED-7* pathway. Based on these data, we conclude that *GULP1* and the *CED-1*, *CED-6* and *CED-7* pathway are involved in schizophrenia. This finding is significant that it opens the door for focused biological studies of the role of these genes in schizophrenia.

There may be ethnic heterogeneity at the rs2004888 locus. Four of the 6 samples used in this study are Caucasian and these samples identify the same risk allele in both family and case-control samples. Collectively, these 4 Caucasian samples produced a highly significant association (*p* = 0.0001, data not shown). In contrast, the combined Chinese samples were not significant. We compared the allele frequency between the Caucasian and Chinese samples, while the major alleles are the same in both Caucasian and Chinese samples, its frequency in the Caucasian sample is significantly lower than that of the Chinese sample (0.878 vs. 0.927). Since the two Chinese samples were not typed for other SNPs in this region, we compared the haplotype structure of *GULP1* between Caucasian and Chinese populations using the SNPs typed in the dbSNP database. In both populations, rs2004888 is in high LD with other markers in this region. However, the frequency of the undertransmitted haplotype in the Caucasian population is significantly higher than that in the Chinese population (0.142 vs. 0.039). These differences in LD structure may be responsible for the differences in association signal observed at this locus.

We have noticed that the OR at rs2004888 is close to some promising candidates recently identified from genome-wide association studies [42], [43]. The *ZNF804A* gene (rs1344706) has an OR of 1.12 and rs17101921 (near *FGFR2* gene) has an OR of 1.17. At rs2004888, we have an OR of 1.25 for the Caucasian case-control samples and an odds of transmission ratio of 1.17 in the family samples. Consistent with these other studies, our results clearly suggest that large samples are necessary to identify these risk genes. This may explain why some individual samples did not produce significant results.

The statistical interactions observed in this study are supported by physical contact and biological modulation. In one study, *ABCA1* protein is found to physically interact with *MEGF10* protein and functionally modulates the *MEGF10*-mediated engulfment of apoptotic cells [16]. In addition to phagocytosis, both *ABCA1* and *ABCA7* are involved in lipid metabolism and homeostasis [64], and there is evidence that these genes are regulated in a coordinated fashion [18]. We also notice that multiple polymorphisms in the *ABCA1* gene have been found to be associated with Alzheimer's disease [11], [21], [49], [61], although the relationship between the Alzheimer disease and schizophrenia remains unclear.

At this time, we don't understand the mechanism how the apoptotic engulfment pathway contributes to schizophrenia. Based on the functions of this pathway, we can speculate how the apoptotic clearance and engulfment pathway might be involved in the disease. In the development of the central nervous system, programmed cell death, i.e. apoptosis, is evident in shaping the neuronal circuitries and functions [25], [31], [40]. Rapid clearance of cell corpses is essential for maintaining tissue homeostasis and preventing the release of potentially cytotoxic or antigenic molecules from dying cells. Defects in cell corpse clearance are closely associated with autoimmune and inflammatory responses in *C. elegans* [50]. There are reports that there are dysfunctions in autoimmune system and elevated amount of inflammatory cytokines in schizophrenia patients [20], [41], [45], [52], suggesting that autoimmune and inflammatory cytokines may be involved in the disease. The defects in the apoptotic pathway may be a factor contributing to dysfunctional autoimmune system and elevated inflammatory cytokines observed in schizophrenia patients. Specific to the genes, mutations in *CED-1*, *CED-6* and *CED-7* results in abnormal commisure patterns [51] and axon pruning [1], [30] in *C. elegans* and *Drosophila*. Since the genes are highly conserved, it would not be a surprise that mutations in their human orthologs (i.e. *MEGF10*, *GULP1*, *ABCA1* and *ABCA7*) would cause similar abnormal projections and synaptic connections.

In this study, we provide genetic evidence that the *GULP1* gene and the apoptotic engulfment pathway may be involved in schizophrenia. Since the engulfment pathway is evolutionarily conserved, many genetic and functional studies in model organisms can provide new insights to understand the pathophysiology of schizophrenia. Following the guidance of these animal model studies, it would be of great interest to examine the functions of these genes in humans and to determine how the dysfunction of these genes may lead to the disease.

## Materials and Methods

### Sample description

#### Ethics statement

This study was conducted according to the principles expressed in the Declaration of Helsinki. The study was approved by Institutional Review Boards/Ethics Committees at Virginia Commonwealth University (ISHDSF and ICCSS), University of Pittsburgh (the Pittsburgh sample), Western Australian University (the German sample), the Zoology Institute of Chinese Academy of Science (Kunming sample) and Jiaotong University (Shanghai sample). All patients provided written informed consent for the collection of samples and subsequent analysis.

#### The ISHDSF sample

The Irish study of high density schizophrenia families (ISHDSF) was collected in Northern Ireland, United Kingdom and the Republic of Ireland. Phenotypes were assessed using DSM-III-R. The diagnoses were originally classified a hierarchy of 10 categories reflecting the probable genetic relationship of these syndromes to classic schizophrenia. In this study, we used the narrow disease definition, which include only categories D1 and D2 in the original classification. In this narrow definition, only patients met DSM-III-R definitions of schizophrenia, poor-outcome schizoaffective disorder and simple schizophrenia were classified as affected. The sample contained 273 pedigrees and about 1350 subjects had DNA sample for genotyping. Of them, 515 were diagnosed as affected using definition described above. Detailed descriptions of the sample have been published previously [22].

#### The ICCSS sample

The Irish case-control study of schizophrenia (ICCSS) sample was collected in the same geographic regions as that of the ISHDSF sample. The affected subjects were selected from in-patient and out-patient psychiatric facilities in the Republic of Ireland and Northern Ireland. Subjects were eligible for inclusion if they had a diagnosis of schizophrenia or poor-outcome schizoaffective disorder by DSM-III-R criteria, and the diagnosis was confirmed by a blind expert diagnostic review. Controls, selected from several sources, including blood donation centers, were included if they denied a lifetime history of schizophrenia. However, the controls were not screened by clinicians. Both cases and controls were included only if they reported all four grandparents as being born in Ireland or the United Kingdom. Using these criteria, a total of 1417 subjects (625 affected subjects and 792 controls) were included in this study.

#### The German family sample

The German sample consisted of two subsamples, 79 affected sib pairs with parents and 125 proband-parents trios. Of the 79 sib pairs, 54 were used in the linkage study where overlapping linkage peak was found between the ISHDSF and German sib samples [53], [57]. The trios were selected from a larger trio sample [44] with positive family history (defined as at least one first or second degree relative of the proband meeting the DSM IV criteria of schizophrenia or schizoaffective disorder).

#### Pittsburgh family sample

The Pittsburgh sample contained 247 nuclear families (case and parents) with a total of 729 subjects with DNA for genotyping. Subjects were recruited from inpatients and outpatients facilities within a 500 mile radius of Pittsburgh and met DSM IV criteria of schizophrenia or schizoaffective disorder. All probands self-reported as with Caucasian ancestry [10].

#### The Shanghai case control sample

The Shanghai case control sample consisted of 942 unrelated patients with schizophrenia and 946 control individuals. All subjects were of Han Chinese origin. A clinical interview was administered by two independent senior psychiatrists to all patients according to the criteria of the DSM-IV. All patients were policlinic and recruited from the Shanghai Mental Health Center, East China. The healthy controls were drawn from the general population in the East China. None had a history of psychotic disorders. Participants were fully informed of, and gave written consent for, the genetics analysis, which was reviewed and approved by the Shanghai Ethics Committee of Human Genetic Resources.

#### The Kunming case control sample

The Kunming case control sample consisted of 516 schizophrenia patients and 794 normal controls. All affected subjects were self-reported as Han Chinese and were recruited from Yunnan Mental Health Hospital, Kunming city, Yunnan Province, China. The patients were diagnosed by two trained psychiatrists independently and met the criteria of the Diagnostic and Statistical Manual of Mental Disorders, 4th edition (DSM-IV) for schizophrenia. Subjects with substance-induced psychotic disorders, learning disabilities, head injuries and other symptomatic psychoses were excluded. The controls were recruited from the same province, and all were self-reported as Han Chinese.

### Marker selection and genotyping

We used the HapMap data (http://www.hapmap.org/) and the available assays developed by Applied BioSystems to assist in our selection of markers. For each gene, we decided to cover the transcribed genomic region plus 50 kb on both ends. We downloaded the Caucasian HapMap data from HapMap website, and analyzed the LD structure with the HaploView program [3]. We selected SNPs (r^2^> = 0.80) tagging major haplotypes (with frequency > = 5%) for each gene. SNPs tagging minor haplotypes (those with frequencies less than 5%) were not considered. The main reasons we considered only major haplotypes were twofold. First, minor haplotypes with low frequencies are normally less reliable because computational inference usually does not do a very good job when the allele frequencies are low. Second, given the sample size of our Irish samples, it is not very likely that we have sufficient power to detect these low frequency haplotypes. Based on these criteria, we obtained 10 SNPs for *GULP1*, 11 SNPs for *ABCA7* and 5 SNPs for *ABCA7*.

Five samples used in this study (ISHDSF, ICCSS, the German, the Pittsburgh and the Shanghai samples) were typed with the TaqMan method [29]. The assays used were either validated assays or custom designed assays developed by Applied BioSystems Corporation (Foster city, CA). Genotypes were scored with a semi-automated procedures developed in our lab [65] or with software from the commercial providers. The Kunming sample was typed with single base extension with capillary electrophoresis. The PCR primers used were TTTTGGATTCGGCGGATTAGG and CTGGAAGTTCGCTCCTGGGTC, and the extension primer used was TTTTTTTTTTTTACCTTACCGCCCCTCGGGATATCAGCTTCT. All markers typed were checked for deviation from the Hardy-Weinberg Equilibrium (HWE) and Mendelian errors by the PEDSTATS program [66]. Detail information of these typed markers in the ISHDSF and ICCSS was listed in Table S1 in the supplementary materials.

### Statistical analyses

#### Single marker association tests

We used the pedigree disequilibrium test (PDT) [34] as implemented in the UNPHASED program (version 2.4, PDTPHASE module) [13] to analyze the ISHDSF sample. In these analyses, both vertical and horizontal transmissions were included. The *p* values reported were based on weighing all families equally (the ave option in the program). For the other 5 individual samples and the combined samples, the newer version of the UNPHASED program (version 3.11), which was designed to analyze case-control samples, family samples or combined case-control and family samples [14], was used to analyze single marker associations. In this analysis, ethnicity was used as a covariate. Meta-analyses were conducted using the Comprehensive Meta-Analysis software 2.0 from Biostat (Englewood, NJ, USA) (www.meta-analysis.com/). We used the HaploView program (version 4.0) [3] to estimate pairwise LD and to illustrate haplotype blocks. The haplotype blocks were partitioned by the confidence interval algorithm [15].

#### Gene-gene interaction

The multifactor dimensionality reduction pedigree disequilibrium test (MDR-PDT) [35] was used to explore multi-locus associations in the ISHDSF sample. The MDR-PDT was a within-family measure of indirect or direct association between genotype and disease. As described previously [35], the PDT statistic [33] functioned within the framework of the MDR algorithm. Genotypes were classified as high and low-risk by comparing the PDT statistic to a threshold of 0, where positive statistics indicate evidence for association at that genotype. The MDR-PDT statistic was then calculated for the pooled high-risk genotypes for each set of loci. The models were ordered and evaluated by MDR-PDT statistics. A permutation test was applied to estimate the significance of the result, which inherently adjusted for the size of the search performed. The permutation test consisted of randomizing status for offspring, holding the proportion of affected individuals constant across permutations, calculating the statistic, and repeating many times to estimate the distribution of the null hypothesis. The test based on the permutation procedure would have the correct type I error rate, even for sparse data. This validity was due to all contingency table cells from each permutation containing the same number of observations as those from the unpermuted data. In this study, we limited our search to include only 2- and 3-locus interactions. To estimate the degree of effect modification between the MDR-PDT model SNPs, we fitted conditional logistic regression models with adjustment for residual correlation among affected offspring due to linkage [54]. For model fitting, the genotypes were specified as high or low-risk, denoted as exposed (1) or unexposed (0) respectively, by MDR-PDT analysis of individual loci.

For the ICCSS sample, we used the multifactor dimensionality reduction method (MDR) [37], [47], [48] to examine potential gene-gene interactions. MDR was a nonparametric method that performs an exhaustive search of all possible interactions and maps the data into a single dimension relevant to association. Similar to the MDR-PDT procedure, significance was evaluated via permutation testing, which inherently adjusted for the multiple comparisons from the search performed. As in the family sample, we limited our search to 2- and 3-locus interactions. The effect of interaction was estimated by generalized linear regression model as implemented in the SPSS software (SPSS for Windows, version 16.0).

## Supporting Information

Figure S1 A comparison of LD between the ISHDSF and ICCSS samples for GULP1, ABCA1 and ABCA7 zgenes.
(0.51 MB DOC)

Table S1 Marker information.
(0.07 MB DOC)

Table S2 Single marker association analyses in the ABCA1 and ABCA7 genes.
(0.06 MB DOC)
