# Supplementary figures and images for: Apoptotic Engulfment Pathway and Schizophrenia
Source: PLoS One. 2009 Sep 1;4(9):e6875. doi: 10.1371/journal.pone.0006875 (PMC2731162; doi:10.1371/journal.pone.0006875)

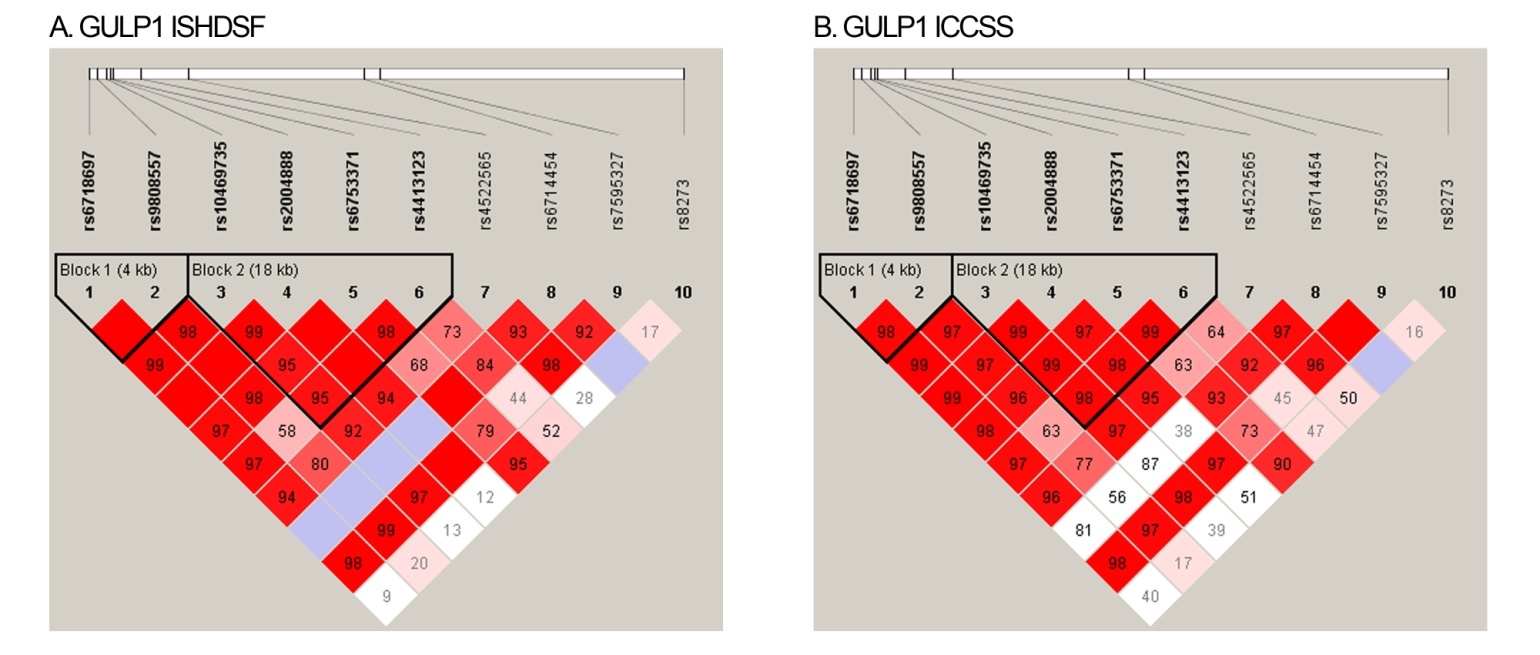


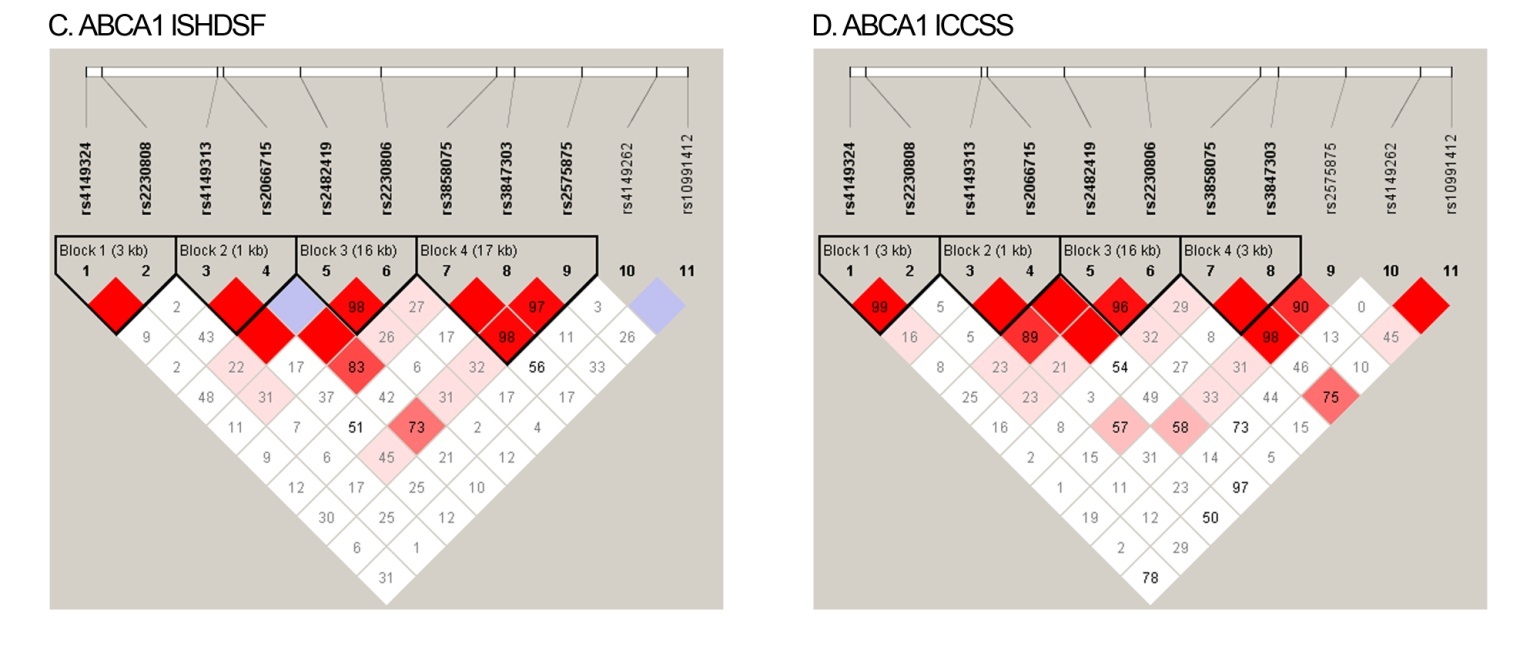


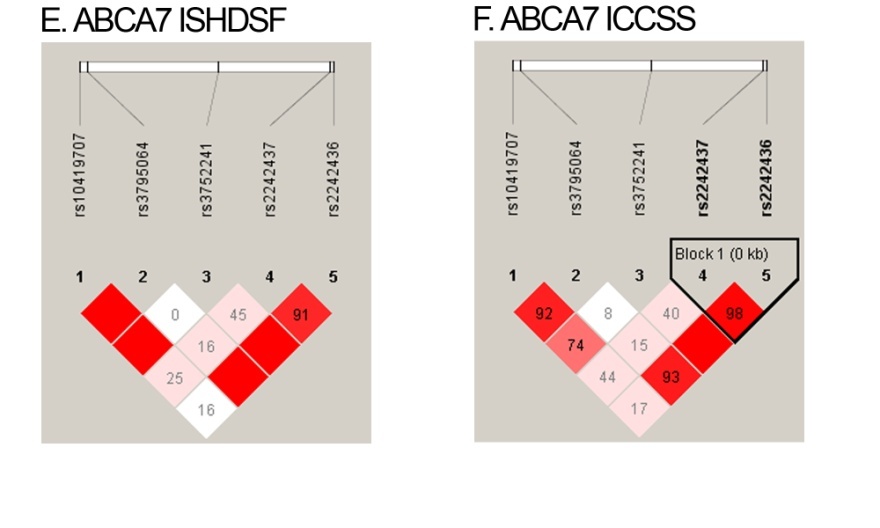


Figure S1. A comparison of LD between the ISHDSF and ICCSS samples for *GULP1*, *ABCA1* and *ABCA7* genes.

Supplement: Figure S1 — A comparison of LD between the ISHDSF and ICCSS samples for GULP1, ABCA1 and ABCA7 zgenes. (0.51 MB DOC) [file pone.0006875.s001.doc]
