# Supplementary material for: Apoptotic Engulfment Pathway and Schizophrenia
Source: PLoS One. 2009 Sep 1;4(9):e6875. doi: 10.1371/journal.pone.0006875 (PMC2731162; doi:10.1371/journal.pone.0006875)
Supplement: Table S1 — Marker information. (0.07 MB DOC) [file pone.0006875.s002.doc]

Table S1. Marker information

|  |  |  |  |  | **ISHDSF** | | **ICCSS** | |
| --- | --- | --- | --- | --- | --- | --- | --- | --- |
| **Gene** | **rs#** | **Chr#:Position** | **SNP (major/minor)** | **Function** | **MAF** | **HWE** | **MAF** | **HWE** |
| GULP1 | rs6718697 | Chr2:188853636 | A/T |  | 0.140 | **0.0233** | 0.117 | 1.000 |
| GULP1 | rs9808557 | Chr2:188857848 | C/T |  | 0.181 | 0.4660 | 0.150 | 0.750 |
| GULP1 | rs10469735 | Chr2:188863010 | T/C |  | 0.144 | 0.3446 | 0.120 | 1.000 |
| GULP1 | rs2004888 | Chr2:188864958 | T/G |  | 0.142 | 0.4142 | 0.121 | 0.707 |
| GULP1 | rs6753371 | Chr2:188866764 | G/A |  | 0.495 | 0.3529 | 0.492 | **0.040** |
| GULP1 | rs4413123 | Chr2:188881292 | G/C |  | 0.469 | 0.0918 | 0.465 | 0.057 |
| GULP1 | rs4522565 | Chr2:188906332 | T/C |  | 0.156 | 0.0912 | 0.143 | 0.149 |
| GULP1 | rs6714454 | Chr2:188999805 | G/A |  | 0.020 | 1.0000 | 0.020 | 0.185 |
| GULP1 | rs7595327 | Chr2:189007663 | G/A |  | 0.199 | 0.5752 | 0.172 | 0.814 |
| GULP1 | rs8273 | Chr2:189168548 | C/T |  | 0.128 | 0.5219 | 0.129 | 0.328 |
| ABCA1 | rs4149324 | Chr9:106599300 | A/G |  | 0.065 | 0.7988 | 0.069 | 0.882 |
| ABCA1 | rs2230808 | Chr9:106602625 | G/A | Arg > Lys | 0.207 | 0.3474 | 0.229 | **0.017** |
| ABCA1 | rs4149313 | Chr9:106626574 | A/G | Ile > Met | 0.120 | 0.3588 | 0.123 | 0.959 |
| ABCA1 | rs2066715 | Chr9:106627854 | G/A | Val > Ile | 0.057 | 0.8753 | 0.061 | 0.475 |
| ABCA1 | rs2482419 | Chr9:106643863 | T/C |  | 0.103 | 0.5579 | 0.106 | 0.760 |
| ABCA1 | rs2230806 | Chr9:106660688 | G/A | Arg > Lys | 0.298 | **0.0041** | 0.291 | 0.786 |
| ABCA1 | rs3858075 | Chr9:106684651 | C/T |  | 0.229 | 1.0000 | 0.219 | 0.566 |
| ABCA1 | rs3847303 | Chr9:106688473 | G/A |  | 0.105 | 1.0000 | 0.132 | 0.620 |
| ABCA1 | rs2575875 | Chr9:106702315 | G/A |  | 0.368 | 0.8307 | 0.379 | 0.076 |
| ABCA1 | rs4149262 | Chr9:106717753 | T/A |  | 0.057 | 0.9065 | 0.064 | 1.000 |
| ABCA1 | rs10991412 | Chr9:106724226 | G/A |  | 0.081 | 0.8799 | 0.079 | 1.000 |
| ABCA7 | rs10419707 | Chr19:989445 | T/C |  | 0.104 | 0.1442 | 0.106 | 0.750 |
| ABCA7 | rs3795064 | Chr19:990401 | C/G |  | 0.189 | 0.4614 | 0.195 | 0.124 |
| ABCA7 | rs3752241 | Chr19:1004524 | C/G | Leu > Leu | 0.181 | 0.3158 | 0.183 | **0.005** |
| ABCA7 | rs2242437 | Chr19:1016563 | G/C |  | 0.264 | 0.1383 | 0.273 | 0.203 |
| ABCA7 | rs2242436 | Chr19:1016945 | G/A |  | 0.201 | 1.0000 | 0.176 | 0.631 |
