# Supplementary material for: Apoptotic Engulfment Pathway and Schizophrenia
Source: PLoS One. 2009 Sep 1;4(9):e6875. doi: 10.1371/journal.pone.0006875 (PMC2731162; doi:10.1371/journal.pone.0006875)
Supplement: Table S2 — Single marker association analyses in the ABCA1 and ABCA7 genes. (0.06 MB DOC) [file pone.0006875.s003.doc]

Table S2. Single marker association analyses in the *ABCA1* and *ABCA7* genes

|  |  | **ISHDSF** | | | | | | | | | **ICCSS** | | | |
| --- | --- | --- | --- | --- | --- | --- | --- | --- | --- | --- | --- | --- | --- | --- |
| **Gene** | **Marker** | **Transmitted Allele** | **Z** | **P** | **Trio-T** | **Trio-NT** | **AffSib** | **UnafSib** | **Allele Freq** | **T/NT** | **P** | **Case Freq** | **Ctrl Freq** | **OR** |
| ABCA1 | rs4149324 | G | 0.228 | 0.8201 | 17 | 8 | 86 | 77 | 0.066 | 1.21 | **0.0258** | 0.083 | 0.061 | 1.40 |
| ABCA1 | rs2230808 | G | 0.478 | 0.6328 | 130 | 134 | 1072 | 1067 | 0.790 | 1.00 | 0.7795 | 0.771 | 0.776 | 0.97 |
| ABCA1 | rs4149313 | A | 0.304 | 0.7609 | 149 | 142 | 1202 | 1199 | 0.879 | 1.01 | 0.6636 | 0.881 | 0.875 | 1.05 |
| ABCA1 | rs2066715 | G | 0.489 | 0.6250 | 158 | 154 | 1316 | 1322 | 0.942 | 1.00 | 0.3050 | 0.935 | 0.945 | 0.84 |
| ABCA1 | rs2482419 | T | 0.428 | 0.6684 | 150 | 147 | 1247 | 1248 | 0.893 | 1.00 | 0.7542 | 0.897 | 0.893 | 1.04 |
| ABCA1 | rs2230806 | A | 0.570 | 0.5687 | 48 | 42 | 432 | 453 | 0.311 | 0.97 | 0.7199 | 0.300 | 0.294 | 1.03 |
| ABCA1 | rs3858075 | C | 0.554 | 0.5800 | 124 | 126 | 1054 | 1041 | 0.763 | 1.01 | 0.6008 | 0.770 | 0.779 | 0.95 |
| ABCA1 | rs3847303 | G | 0.074 | 0.9409 | 141 | 136 | 1210 | 1206 | 0.891 | 1.01 | 0.6996 | 0.873 | 0.868 | 1.05 |
| ABCA1 | rs2575875 | G | 0.538 | 0.5904 | 97 | 92 | 872 | 845 | 0.620 | 1.03 | 0.6076 | 0.612 | 0.622 | 0.96 |
| ABCA1 | rs4149262 | A | 0.354 | 0.7232 | 16 | 11 | 77 | 83 | 0.053 | 0.99 | 0.7392 | 0.066 | 0.063 | 1.05 |
| ABCA1 | rs10991412 | G | 0.905 | 0.3657 | 151 | 153 | 1273 | 1257 | 0.915 | 1.01 | 0.6416 | 0.930 | 0.925 | 1.07 |
| ABCA7 | rs10419707 | T | 0.14 | 0.8853 | 151 | 144 | 1218 | 1220 | 0.899 | 1.00 | 0.3694 | 0.900 | 0.889 | 1.12 |
| ABCA7 | rs3795064 | G | 1.01 | 0.3139 | 37 | 33 | 263 | 248 | 0.174 | 1.07 | 0.4303 | 0.202 | 0.189 | 1.08 |
| ABCA7 | rs3752241 | G | 0.29 | 0.7693 | 29 | 21 | 226 | 194 | 0.169 | 1.19 | 0.6516 | 0.183 | 0.176 | 1.05 |
| ABCA7 | rs2242437 | C | 1.09 | 0.2779 | 40 | 38 | 338 | 304 | 0.279 | 1.11 | 0.9469 | 0.266 | 0.265 | 1.01 |
| ABCA7 | rs2242436 | G | 3.22 | **0.0013** | 130 | 122 | 1135 | 1069 | 0.809 | 1.06 | 0.4286 | 0.825 | 0.813 | 1.08 |
